# An overview of insights and updates on TTN mutations in cardiomyopathies

**DOI:** 10.3389/fphar.2025.1668544

**Published:** 2025-10-20

**Authors:** Sarmistha Saha, Gazmend Temaj, Pelin Telkoparan-Akillilar, Silvia Chichiarelli, Luciano Saso

**Affiliations:** ^1^ Department of Biotechnology, Institute of Applied Sciences and Humanities, GLA University, Mathura, Uttar Pradesh, India; ^2^ Faculty of Pharmacy, College UBT, Prishtina, Albania; ^3^ Department of Medical Biology, Faculty of Medicine, Gazi University, Ankara, Türkiye; ^4^ Department of Biochemical Sciences “A. Rossi-Fanelli”, Sapienza University of Rome, Rome, Italy; ^5^ Department of Physiology and Pharmacology “Vittorio Erspamer”, La Sapienza University, Rome, Italy

**Keywords:** titin, TTN, titinopathies, cardiomyopathy, cardiovascular genetics

## Abstract

Within the heart muscle, the largest sarcomeric protein is titin (TTN). The heart expresses two principal isoforms, N2B and N2BA, which arise from alternative splicing of the TTN gene. These isoforms span four distinct regions of the sarcomere: the Z-line, I-band, A-band, and M-line. Titin, encoded by the extensive TTN gene consisting of 364 exons, plays a critical role in the structural integrity, development, mechanical properties, and regulation of both cardiac and skeletal muscles. The purpose of this review is to provide a comprehensive understanding of the critical role TTN mutations play in DCM and other forms of cardiomyopathy. With the advent of next-generation sequencing (NGS), it has become feasible to simultaneously analyse numerous genes, including large and complex ones such as TTN. TTN truncations are frequently observed in dilated cardiomyopathy (DCM), whereas they are comparatively rare in hypertrophic cardiomyopathy (HCM). Furthermore, TTN mutations have been implicated in arrhythmogenic right ventricular cardiomyopathy (ARVC), a distinct clinical entity with characteristic features and outcomes. The discovery of a rare TTN missense variant that co-segregates with restrictive cardiomyopathy (RCM) strongly suggests that TTN may represent a novel causative gene in this severe cardiomyopathy. Furthermore, we highlight the significant implications of these findings for advancing both basic research and clinical practice in cardiovascular medicine.

## 1 Introduction

**FIGURE 1:**
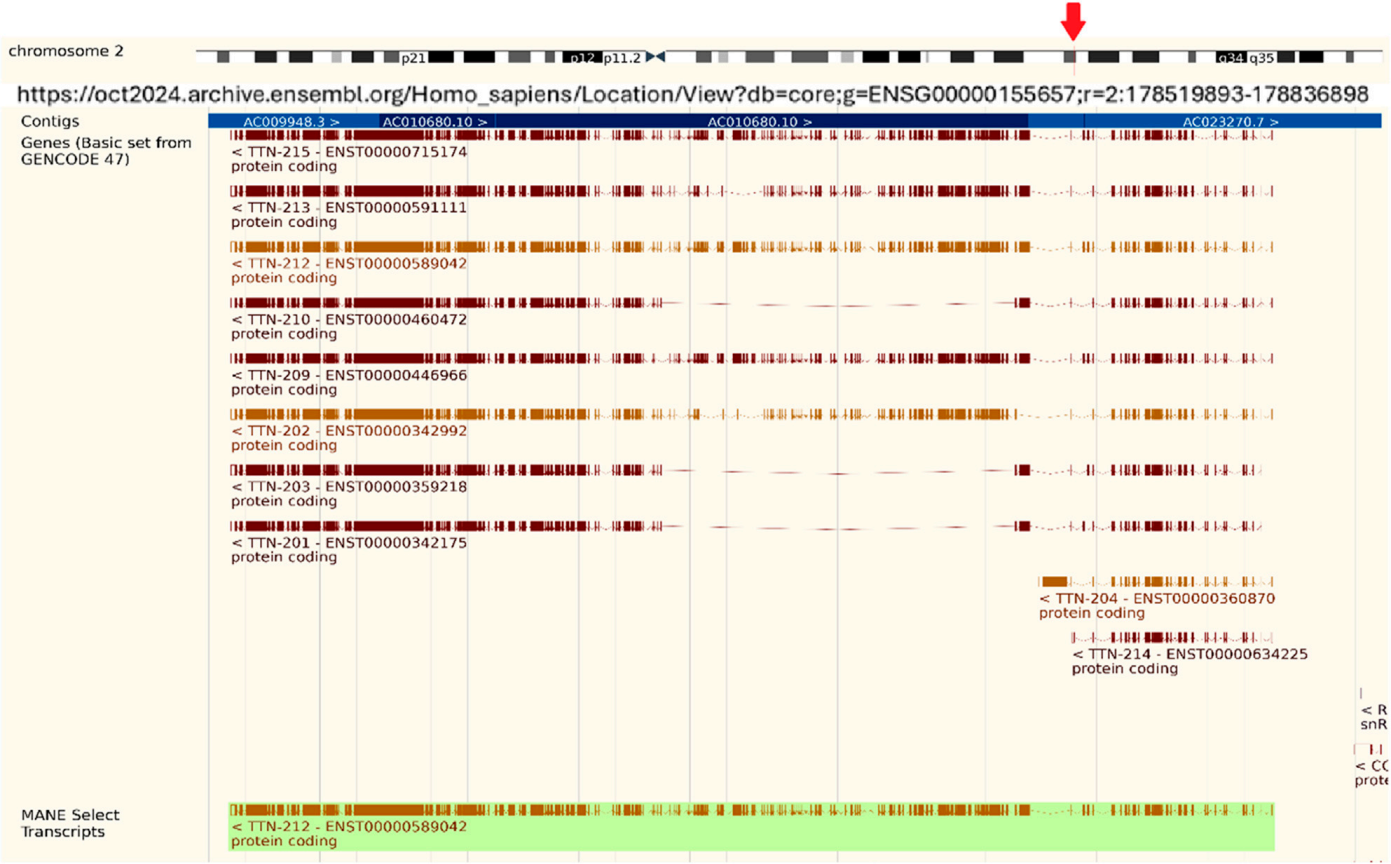
Titin Gene (TTN) information. Titin Gene (TTN) is located on chromosome 2, spanning 304,814 bps from 178,525,989 to 178,830,802. It has multiple transcripts, containing a total of 376 exons on the reverse strand, and includes 117,653 SNPs. (Source: https://oct2024.archive.ensembl.org/Homo_sapiens/Location/View?db=core; g=ENSG00000155657;r=2:178519893-178836898).

The fundamental structural component that makes striated muscle contraction easier is the sarcomere. The large filamentous protein titin (TTN) is a key component of the sarcomere. The TTN gene contains 364 exons, located on chromosome 2q31, that encode the largest known protein in the human body with a molecular weight of ≤4 mDa and a length of 27,000–33,000 amino acids (Figure 1) (Cazorla et al., 2000; Li et al., 2020). TTN spans half of the sarcomere, connecting the Z-disk to the M-line and functions structurally as a biological spring.

TTN is composed of four segments: The N-terminal region that anchors and embeds TTN to the sarcomere Z-disk is known as the Z-line. The main spring-like elements in the I-band are the unstructured regions of N2B and the PEVK region. Only when these spring elements are extended do Ig-like domains start contributing to passive tension. The rigid A-band, composed of alternating immunoglobulin-like (Ig) regions and fibronectin domains, serves as a stable anchoring site for myosin filaments, facilitating their effective binding and force generation during muscle contraction. Titin is tethered to the thick filament by wrapping around the myosin tails, with its three C-terminal domains (C8–10) binding strongly to the myosin tail and running axially along the thick filament. This positioning enables titin not only to serve as a structural scaffold but potentially to interact with the myosin heads, contributing to the regulation of thick filament stability and force generation during muscle contraction (Squarci et al., 2023; Herzog, 2018). Beyond its architectural role, TTN is crucial for sarcomere assembly, mechanosensing, and signal transduction. A distinct feature of TTN is its extensive alternative splicing, which results in three main isoforms: N2A, N2B, and N2BA (Kellermayer et al., 2019). These isoforms mostly differ in the length and elasticity of their I-band regions. In the adult cardiomyocytes, two different isoforms, N2B and N2BA, are predominantly expressed. N2BA is longer and more compliant, whereas N2B is shorter and stiffer, contributing differently to myocardial elasticity (Fomin et al., 2021). TTN mutations have been strongly associated with cardiac diseases, especially dilated cardiomyopathy (DCM), which is characterized by systolic dysfunction and ventricular enlargement in the absence of identifiable causes such as ischemic, congenital, hypertensive, or valvular etiologies (de Lavallaz et al., 2023).

Similar to hypertrophic cardiomyopathy (HCM) in the 1990s, dilated cardiomyopathy (DCM) has recently been shown to have a significant hereditary component. Left ventricular enlargement (LVE) and systolic dysfunction with an ejection fraction (EF) of 50% (Huggins et al., 2022) or, more strictly, 45% (Burkett and Hershberger, 2005) are characteristics of DCM. Among all causes of heart failure in the United States, DCM accounts for at least half of the cases. Heart failure syndrome is characterized by insufficient cardiac output to sustain the body’s circulation and nutrition. According to the 2010 American Heart Association, 5.8 million Americans suffered from heart failure, and a sizable percentage of them were diagnosed with DCM of unknown etiology, often known as idiopathic DCM (IDC) (Siddiqi et al., 2022; Lindberg et al., 2025; Wang et al., 2025). A genetic basis has been identified in approximately 30%–35% of IDCs (in familial or seemingly sporadic cases), whether familial or sporadic. This discovery enables genetic testing, which has become increasingly accessible and cost-effective. Moreover, DCM presymptomatic therapies are effective in reducing morbidity and death.

TTN truncation mutations are the most common genetic cause of DCM. Familial TTN variants are responsible for approximately 25% of DCM cases, while sporadic variants account for about 18% (LeWinter and Granzier, 2013). Although it is not yet fully established that TTN truncating mutations are generally harmful, studies suggest they might be (Akinrinade et al., 2015). Genotype–phenotype correlations in TTN-related cardiomyopathies reveal that mutations in different titin regions distinctly impact disease penetrance, severity, and outcomes. Truncating variants in the A-band, which corresponds to constitutively expressed exons with high proportion spliced-in (PSI) scores, exhibit a stronger association with dilated cardiomyopathy (DCM) and higher odds ratios for disease than variants in regions such as the I-band and Z-disk, which are alternatively spliced with lower PSI values. Mutations in the I-band may be partially mitigated by exon skipping, reducing pathogenicity. Additionally, emerging evidence indicates the location of variants influences clinical outcomes, with A-band TTNtv linked to more severe contractile dysfunction and increased arrhythmia risk (Kim et al., 2023). Advances in genomic sequencing, bioinformatic pathogenicity predictors (including PSI scoring), and clinical imaging such as cardiac MRI are increasingly essential for distinguishing variants of uncertain significance (VUS) from clinically actionable mutations. These integrative approaches refine interpretation accuracy, enabling personalized risk assessment and management in TTN cardiomyopathy patients. Notably, truncations in the A-band region of TTN are responsible for up to 25% of DCM cases (Granzier and Irving, 1995). Beyond DCM, TTN mutations also contribute to the pathophysiology of other cardiomyopathies, including restrictive cardiomyopathy (RCM) and arrhythmogenic right ventricular cardiomyopathy (ARVC), which is believed to be a genetic condition with 30%–50% of cases being familial (Han et al., 2025; León et al., 2024; Micaglio et al., 2021). Additionally, TTN is implicated in hypertrophic cardiomyopathy (HCM). The pathogenic mechanism of titin truncating variants (TTNtv) in DCM is likely at the RNA level rather than via abundant truncated protein production which is supported by several recent studies (Schafer et al., 2017; Kellermayer et al., 2024). These findings suggested that most TTNtv transcripts undergo nonsense-mediated mRNA decay (NMD), reducing expression of truncated titin protein, and the heterozygous mice models rarely develop DCM phenotypes unless with second hit stressors.

## 2 Mechanistic studies of mutations in TTN

**FIGURE 2:**
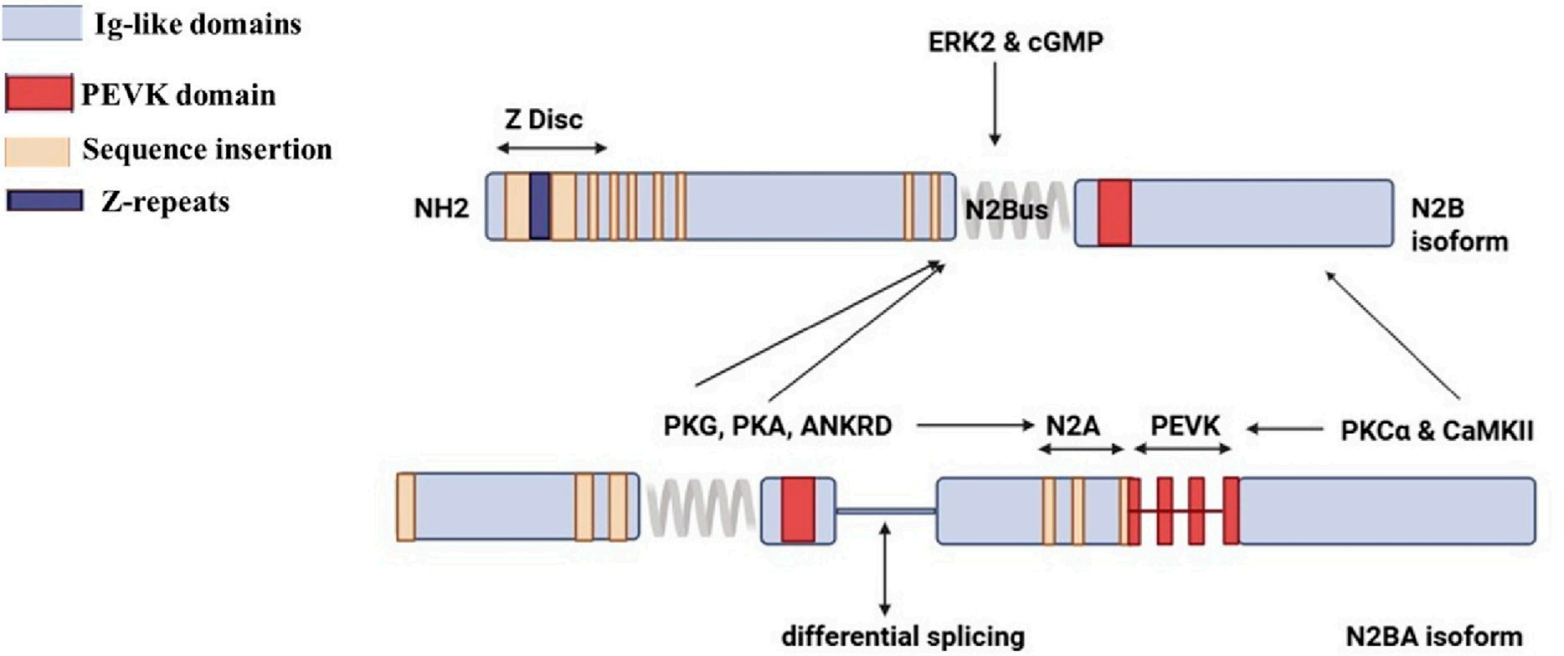
Structure of titin N2B and N2BA isoforms. (Created with BioRender.com).

The TTN isoforms N2BA and N2B comprise approximately 30%–40% and 60%–70%, respectively, of the TTN protein in healthy adult human hearts, maintaining a critical balance that regulates cardiomyocyte stiffness (Hidalgo and Granzier, 2013; Cazorla et al., 2000; LeWinter and Granzier, 2013; Loescher et al., 2022). The N2BA isoform, with its longer, more extensible I-band region, provides greater compliance, whereas the shorter N2B isoform contributes to increased stiffness. The functional significance of TTN I-band regions - particularly N2A and N2B- stems from their rich composition of non-repetitive sequences and immunoglobulin-like (Ig) domains, which serve as scaffolds for multiple protein-protein interactions. Notably, two members of the four-and-a-half LIM domain protein family, DRAL/FHL2 and FHL1, specifically associate with the N2B region (Raskin et al., 2012). Additionally, small heat shock proteins (sHSPs) such as αB-crystallin and HSP27 have binding sites that extend into the N2B region as well as its two COOH-terminal Ig domains (IIg26-IIg27) (Kotter et al., 2014). These sHSPs also interact with N2A elements and the lysine methyltransferase Smyd2 (Donlin et al., 2012). The methylated form of Smyd2-methyl-Hsp90 complex, is essential for stabilizing the N2A and maintaining the integrity of the sarcomeric I-band structure (Donlin et al., 2012; Linke and Hamdani, 2014). Protein kinases A and G (PKA and PKG) also bind to the N2B region, where they facilitate phosphorylation events that reduce TTN passive tension (Figure 2) (van der Pijl et al., 2021; Krüger and Linke, 2006; Kruger et al., 2009). PKG also interacts with the N2A region of TTN (Kruger et al., 2009), along with the skeletal muscle-specific, Ca^2+^-dependent cysteine protease calpain 3/p94. Furthermore, members of the muscle ankyrin repeat protein (MARP) family, including cardiac ankyrin repeat protein (CARP), which is actively involved in regulating the NFκB pathway (Laure et al., 2010), ankrd-2/Arpp, and the diabetes-related ankyrin repeat protein DARP (Hayashi et al., 2008), also demonstrate a binding affinity for the N2A region. This complex interplay of interactions highlights the importance of these regions in cardiac stability and function, providing potential avenues for further research into therapeutic interventions targeting these proteins in heart disease.

The enhanced compliance arises from the additional spring elements found in the proline, glutamate, valine, and lysine-rich region (PEVK) spring elements and tandem Ig regions of N2BA, which are directly linked to reduced passive tension in cardiomyocytes. Among these, the PEVK region plays a critical role in binding interactions with sarcomeric actin (Nagy et al., 2004), calcium ions (Ca^2+^), and calcium-binding protein S100A1. Notably, S100A1 inhibits the binding of actin to the PEVK segment in a calcium-dependent manner and has been observed to engage with multiple sites along TTN’s extensible region *in situ* (Yamasaki et al., 2001). Furthermore, tropomyosin, when in a complex conformation with actin (Raynaud et al., 2004), along with nebulin (Ma and Wang, 2002) and calpain-1 (Raynaud et al., 2005), also binds to the PEVK region. This highlights the multifunctional capacity of the PEVK region and its significance beyond just its interactions within the TTN Z-disk or Z/I junction regions. Calpain 1 (Capn1) can interact with both the PEVK domain and the proximal I-band region of titin. In muscle cells, Capn1 is involved in proteolytic turnover of titin and other sarcomeric proteins. These interaction sites facilitate targeted cleavage and remodeling of titin during muscle adaptation or stress (Lostal et al., 2019). Similarly, Calpain 3 (Capn3), a muscle-specific protease, also binds to titin in the PEVK region. This interaction is critical for regulating Capn3’s proteolytic activity and stability. The PEVK domain contains specific binding sites that modulate Capn3 activity, impacting muscle physiology and disease states such as limb-girdle muscular dystrophy. Alterations in this interaction can lead to abnormal proteolysis and muscle pathology. In essence, Capn1 and Capn3 regulate muscle maintenance and remodeling through their carefully orchestrated binding and cleavage of titin at these specific domains (Lostal et al., 2019). The PEVK region is a regulatory hotspot for calpain interactions affecting protease activity and muscular integrity.

A severe variation of DCM is called RBM20 (RNA binding motif protein 20) cardiomyopathy. A recent research used antisense oligonucleotides (ASOs) to precisely block Rbm20 expression, which downregulated Rbm20 (Methawasin et al., 2025). Their findings showed that the cytoplasmic RNP granules inside the Rbm20S639G cardiomyocytes were considerably reduced when the amount of Rbm20 expression was diminished by treatment with ASOs. When ASO therapy was started prior to the disease’s commencement, the severity of DCM acquired was lessened. Crucially, mice with preexisting DCM showed a marked improvement in ejection fraction and a reduction in the severity of left ventricular chamber dilatation upon starting ASO therapy, which reversed cardiac dysfunction and remodeling. The repair of mis-splicing of RBM20 target genes, including the main target gene *TTN* and other genes like Camk2d, Ryr2, and Ank3, is not necessary for these positive effects to occur (Methawasin et al., 2025).

The relationship between TTN isoform expression and passive myocardial tension is not just a biological detail; it is a cornerstone of cardiac function. A larger elastic I-band region directly correlates with lower passive tension, highlighting the adaptability and responsiveness of heart muscle to physiological demands (Neagoe et al., 2003; Cazorla et al., 2000; Lahmers et al., 2004). Moreover, the variability in isoform expression and TTN splicing is critically important in a range of cardiac diseases. In cases like DCM, there is a striking upregulation of the compliant N2BA isoform, resulting in decreased passive stiffness and greater compliance of the heart chambers ((Neagoe et al., 2003; Cazorla et al., 2000; Opitz et al., 2004). Understanding and manipulating these relationships could pave the way for innovative treatments and improved outcomes in patients with cardiac conditions.

A pivotal study by Roberts et al. (2015) sheds light on the significance of TTN truncating variants (TTNtv). This study demonstrated that both exon usage and the variant’s distance from the protein’s N-terminus play crucial roles in determining their clinical impact. Notably, TTNtv affects exons included in both the N2BA and N2B isoforms are markedly more prevalent in patients with dilated cardiomyopathy (DCM) compared to healthy controls, showing a stronger association with the condition than those affecting only the N2BA isoform. In contrast, control subjects predominantly exhibited truncations from exons not included in the N2BA and N2B transcripts. These insights underline the need for a deeper understanding of TTN variants to improve diagnosis and treatment strategies for DCM. Another review by Granzier and Labeit (2025) showed TTNtv as a major genetic cause of DCM, including mechanistic insights suggesting haploinsufficiency and sarcomere disarray. Important posttranscriptional regulation by factors like RBM20 controlling alternative splicing and resulting isoform shifts that affect myocardial stiffness. They suggested therapeutic interventions by ASOs to modulate splicing and CRISPR-Cas9 gene editing to correct TTN variants.

The M-line region of TTN is uniquely characterized by the presence of a serine-threonine kinase domain (TK) (Tharp et al., 2020), which plays a central role in regulating TTN expression and protein turnover (Lange et al., 2005). Intramolecular interactions with its autoinhibitory tail intricately control this domain, ensuring precise regulation of its function. At the M-line extremity, TTN forms a robust ternary complex with several key structural and functional partners, including DRAL/FHL2 in the Mis2 region (Lange et al., 2002), myomesin at the MIg-4 domain, and M-protein (Fukuzawa et al., 2008). The Binding Integrator-1 (BIN1) interaction site is strategically positioned within the unique Mis4 sequence of TTN (Fernando et al., 2009). Notably, *in vivo* studies have demonstrated that nuclear TTN Mis6 regions engage with lamin (Zastrow et al., 2006), highlighting its significance in cellular structure and function. In addition to its interactions in the I-band, calpain-3/p94 also establishes a critical connection with the M-band portion of TTN. This interaction occurs at a minimal binding site that overlaps the Ig-9 motif (MIg9) and its adjacent unique sequence Mis7, both of which are encoded by the alternatively spliced exon Mex5 (Beckmann and Spencer, 2008). Recently, the cardiomyopathy-associated protein CMYA5 (also known as myospryn) has emerged as an important M-line ligand. Its minimal binding site resides within MIg-10. Remarkably, the entire region from MIg-9 through Mis7 to MIg-10 can facilitate the binding of CMYA5 to TTN, underscoring the complex interactions at play (Sarparanta et al., 2010). Furthermore, the giant protein obscurin and its partner, obscurin-like-1 protein also bind to the ultimate Ig-like domain of TTN (MIg-10) (Pernigo et al., 2010), further reinforcing the critical role of TTN in cardiac function and structural integrity.

The organization of the TTN protein is strategically adapted to support extensive splicing, a key feature that underpins its functional diversity. Roberts et al. (2015) introduced the percentage spliced in (PSI) score, leveraging RNA sequencing data from patients with end-stage dilated cardiomyopathy (DCM) as well as donor hearts. This novel approach provides an invaluable metric for assessing the average usage of each TTN exon, paving the way for deeper insights into their expression patterns. Exons with high PSI values signify those that are constitutively expressed across all TTN isoforms, indicating their essential role in cardiac function. In contrast, exons with lower PSI values typically appear in only one isoform and exhibit reduced expression levels. Remarkably, the link between exon symmetry and PSI reinforces this understanding: of the 175 exons with a PSI below 0.99, a mere three were asymmetric. This contrasts sharply with the 27% of exons above 0.99 that displayed asymmetry, underscoring the predominance of symmetric exons in the TTN gene, over 80%, and suggesting that their exclusion would unlikely disrupt the translational reading frame. For example, in the I-band region, characterized by a lower PSI, an impressive 93% of the alternately spliced exons are symmetric. This indicates that TTNtv in the I-band region is more likely to affect exons that are either spliced out or minimally expressed across most transcripts, thereby significantly reducing their potential to exert harmful effects. Furthermore, it is crucial to understand that while the sequence primarily dictates the stiffness of TTN within the I-band segment of each isoform, cardiac passive tension is also profoundly modulated by post-translational modifications particularly those affecting contractile and regulatory proteins (Yamasaki et al., 2002). Numerous studies have compellingly demonstrated that the phosphorylation of protein kinases can significantly alter the stiffness of the N2B and PEVK spring elements (Bianco et al., 2015). Specifically, the phosphorylation of the N2B spring element by protein kinase A (PKA) and protein kinase G (PKG) results in a marked reduction in passive tension (Belin et al., 2007). This highlights the intricate mechanics of cardiac function and emphasizes the exciting potential for targeted therapeutic interventions that could enhance cardiac performance and resilience.

**FIGURE 3:**
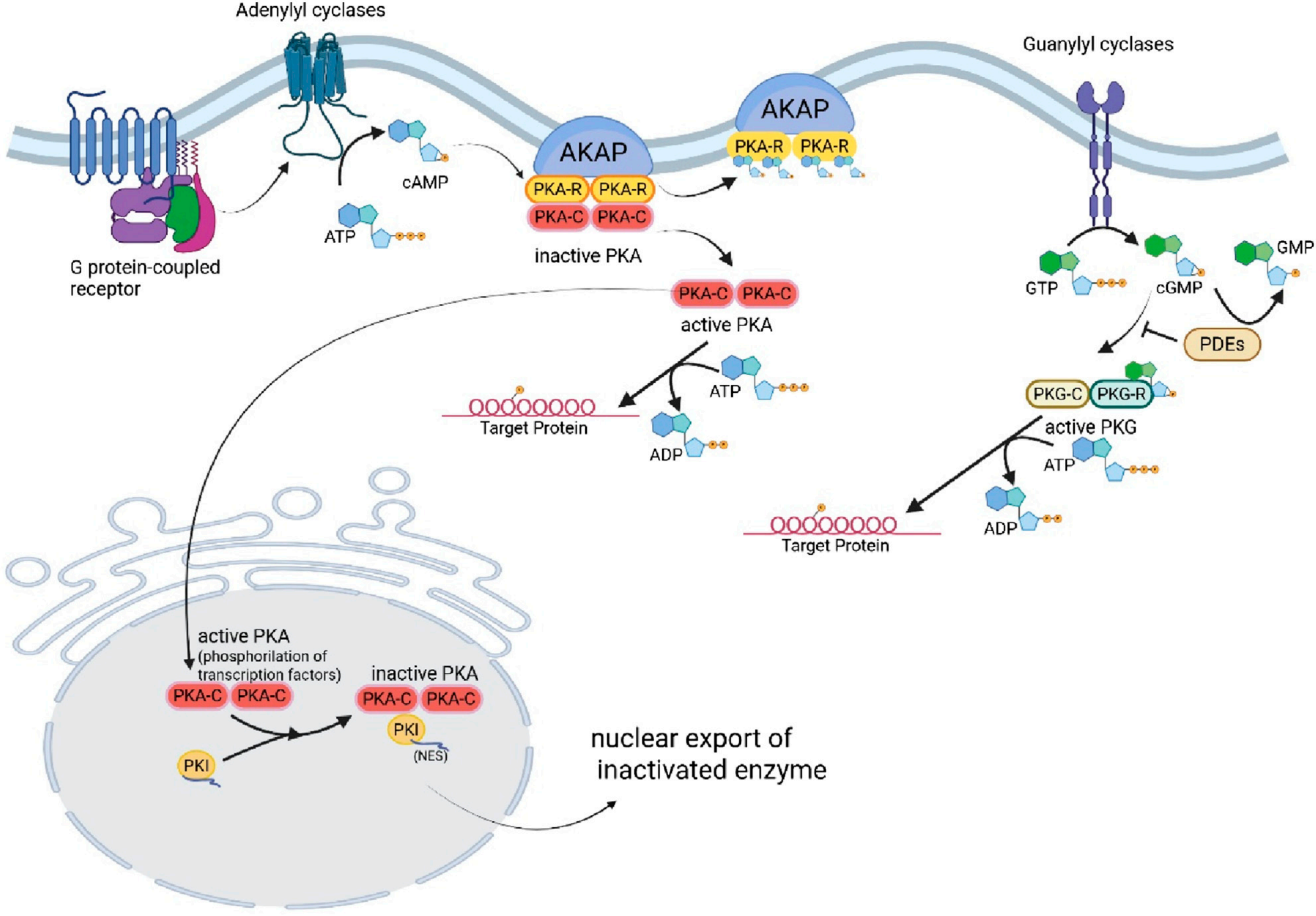
Regulation of PKA and PKG, and Titin phosphorylation. (Created with BioRender.com).

The mechanisms driving changes in TTN isoform expression remain an area of active investigation, yet evidence highlights the crucial role of RNA-Binding Motif Protein 20 (RBM20), a vital RNA splicing factor. A decrease in RBM20 levels can significantly disrupt TTN splicing and isoform expression in both humans and mice (Guo et al., 2012; Methawasin et al., 2014), contributing to the onset of DCM. As a result, the myocardial stiffness associated with TTN is profoundly influenced by the specific composition of TTN isoforms and the phosphorylation state of the elastic I-band. Various kinases impact the elasticity of TTN in distinct ways, underscoring the complexity of this regulation (Figure 3). Importantly, alterations in post-translational modifications, particularly hypophosphorylation, play a crucial role in the pathophysiology of heart disease, emphasizing the need for further research in this critical area (LeWinter and Granzier, 2013).

## 3 Co-relation of TTN with cardiomyopathies

Among the diverse phenotypes associated with TTN mutations, DCM (MIM #604145) remains most clinically significant and well-characterized. To date, 69 distinct TTN mutations have been identified in patients with DCM, further emphasizing the gene’s central role in the pathogenesis of this condition. A study exploring disease-causing variants in DCM has sequenced a substantial cohort of 766 patients, revealing that the TTN gene emerges as the most significant contributor. Astonishingly, nearly 14% of these patients carry at least one mutation in the TTN gene (Pugh et al., 2014). Furthermore, recent discoveries have identified seven distinct TTN mutations - comprising three nonsense mutations, three frameshift mutations, and one missense mutation - in families affected by DCM. This includes patients diagnosed with peripartum cardiomyopathy, underscoring its recognition as a critical initial manifestation of familial DCM (van Spaendonck-Zwarts et al., 2014). These findings highlight the urgent need for further investigation into the implications of TTN mutations in the diagnosis and treatment of DCM.

A comprehensive meta-analysis by Fang and Liu (2020) reveals a significant connection between TTN mutations and familial dilated cardiomyopathy (DCM). Their findings show that patients with familial DCM are more likely to carry TTN mutations than those with sporadic forms of the disease, emphasizing the hereditary nature of these mutations. While the analysis provides compelling evidence, it is important to note that potential biases and confounding factors could not be entirely ruled out.

In a recent compelling study (Schiabor Barrett et al., 2023), the authors harnessed longitudinal electronic health record and exome sequencing data from nearly 450,000 individuals across two major health systems to validate and illuminate the genetic signature of TTNtv-related diagnoses beyond CM, including conditions like atrial fibrillation (Afib). They meticulously focused on TTNtv variants in exons with a splicing percentage exceeding 90% (hiPSI TTNtvs), indicative of strong cardiac expression. Their thorough retrospective analyses uncovered a striking finding: individuals with hiPSI TTNtvs who also experienced early Afib exhibited a remarkable 33% prevalence of CM. This figure far surpasses the prevalence observed in those with TTNtvs without Afib, as well as in individuals without TTNtvs who are diagnosed with early Afib. These groundbreaking results highlight the power of integrating phenotypic information with genomic population screening, demonstrating an effective approach to identify patients at significantly elevated risk of progressing to symptomatic heart failure.

A comprehensive narrative review study by Jolfayi et al. (2024) has revealed a total of 611 distant variants in the TTN gene, meticulously classified into three categories: pathogenic, likely pathogenic, and variants of uncertain significance (VUS). Notably, an impressive 85% of these variants are found within exon fragments, with a striking 69.6% categorized as pathogenic, 21.6% as likely pathogenic, and 8.8% as VUS, according to the rigorous standards set by the American College of Medical Genetics and Genomics (ACMG). Diving deeper into the data, substitutions make up 57.25% of the identified variants, followed by deletions at 29.62%, duplications at 7.36%, and insertions at 5.72%. It is particularly noteworthy that the majority of pathogenic variants are concentrated beyond exon 326, exhibiting elevated CADD (Combined Annotation Dependent Depletion) scores that underscore their clinical significance. Moreover, GERP (Genomic Evolutionary Rate Profiling) scores demonstrate a high degree of conservation among the nucleotides of this gene, with many variants showing substantial GERP scores. Exons at the gene’s terminal end also show higher average CADD scores, while VUS variants consistently reflect lower CADD scores. The TTN gene has a well-documented hotspot for mutations at exon 326, located in the crucial A-band region. Remarkably, exon 358, also in the A-band, exhibits the highest frequency of TTN variants. The concentration of these variants in a select few exons, primarily those in the A- and I-bands, suggests a significant underlying pattern. This distribution pattern may reflect the higher fatality rates associated with mutations in other regions of TTN, which could lead to underrepresentation in clinical cohorts. Alternatively, it may suggest that mutations in certain exons lack clinical symptoms, thus avoiding the need for genetic evaluation. This pattern reinforces the importance of focusing on the A- and I-band exons in understanding TTN-related conditions.

A comprehensive quantitative analysis of genetic variants has compellingly identified exon 326 as the predominant hotspot for variants (2024). Situated in the A band, this exon features the Fibronectin type III domain and displays a remarkably higher frequency of variants than any other region. Exon 358, which encompasses both an Ig-like domain and another Fibronectin type III domain, follows closely with a significant number of variants. Exon 48, while notable, is less prevalent in variant occurrences. Furthermore, among intronic regions, intron 47 emerges as the most significant hotspot for variants, highlighting its importance in the overall variant landscape.

Among the reported TTN mutations linked to DCM, the findings reveal a significant and concerning distribution. A total of 29 mutations are classified as nonsense mutations, with three occurring in the I-band (including two within the critical cardiac-specific N2B exon) and the remaining 26 situated in the A-band. In addition, there are 17 frameshift mutations, with three found in the I-band and an alarming 14 in the A-band. Furthermore, 18 mutations have the potential to disrupt TTN splicing, notably including two situated in the PEVK region, which is essential for cardiac function. Additionally, seven missense mutations are spread throughout the gene, with three in the Z-line, three in the I-band, and one in the Mex1 exon of the M-line. These mutations collectively underscore the critical role of TTN in cardiac health and the profound implications they hold for understanding and addressing DCM. Two specific missense mutations at the Z-line DCM significantly disrupt the binding domains of TTN with α-actinin and T-cap. These alterations critically impair TTN’s ability to interact with these vital partners, as demonstrated by compelling evidence from yeast two-hybrid assays (Itoh-Satoh, et al., 2002).

The c.13108C>T, p. Gln4370 mutation, located within the I-band is strongly predicted to result in the truncation of TTN shortly after the Z-line region. This finding is supported by Itoh-Satoh et al. (2002). The yeast two-hybrid analysis compellingly demonstrated a marked decrease in the binding affinity between TTN and DRAL/FHL2, as evidenced by Matsumoto et al. (2005). This finding suggests that the mutation has significant functional consequences, particularly by disrupting key protein-protein interactions essential for sarcomeric integrity.

In recent developments, CRISPR-Cas9 genome editing has emerged as a tool to successfully correct TTN variations in human induced pluripotent stem cell-derived cardiomyocytes (iPSC-CMs) (Romano et al., 2022). Taking the next vital step, extending these promising results to model organisms such as mice could significantly bolster the therapeutic potential of this innovative approach. In the context of idiopathic DCM, strategically targeting the RBM20 gene presents an exciting avenue for therapy. A substantial number of DCM patients exhibit a detrimental shift in titin isoforms, favoring the longer N2BA isoform, which contributes to decreased ventricular passive stiffness and pronounced systolic dysfunction (LeWinter and Granzier, 2014). By enhancing RBM20 expression through the regulation of transcriptional factors, we could effectively shift the titin isoforms back to favor the N2B isoform. This adjustment holds the promise of restoring titin-based myocardial passive stiffness, potentially transforming the outlook for patients suffering from this condition (Guo et al., 2012). By effectively reducing the expression of RBM20 either through genetic editing or antisense oligonucleotide (ASO)-mediated RNA degradation, researchers have demonstrated compelling evidence that upregulating larger TTN isoforms can significantly improve diastolic function in rodent models of heart failure with preserved ejection fraction (HFpEF) (Radke et al., 2021). It is crucial to understand that RBM20 plays a pivotal role in regulating the splicing of genes associated with calcium (Ca^2+^) handling (Guo and Sun, 2018). When RBM20-dependent splicing of these critical genes is impaired, the heart becomes increasingly vulnerable to arrhythmias (Guo et al., 2012). Consequently, manipulating RBM20 expression to alter titin size could have serious and detrimental effects on cardiac function. Importantly, the consensus sequence UCUU has been identified as the key binding motif for RBM20 within titin mRNA (Maatz et al., 2014). This opens up a promising avenue for therapeutic intervention by designing ASOs that specifically target the RBM20 binding sites on TTN pre-mRNA to reduce diastolic stiffness in patients with heart failure with HFpEF and DCM. This targeted approach could enhance exon inclusion and promote the expression of larger titin isoforms without disrupting the splicing of other essential RBM20 target genes. Moreover, combining these strategies may yield even greater benefits. Given that RBM20 binds at multiple sites on TTN, creating ASOs to inhibit this binding is a complex challenge. However, strategically reducing RBM20 expression has been shown to level of increase the N2BA titin isoform. It is imperative, though, that any reduction in RBM20 expression is meticulously controlled to prevent severe disruptions in the alternative splicing of other mRNA targets, particularly those crucial for calcium handling (Guo et al., 2012). Such a balanced approach could pave the way for innovative therapies that improve heart function and overall patient outcomes.

Hahn et al. (2019) studied the effects of AONs aimed at facilitating precise exon skipping in targeted TTN exons. They selected a well-characterized TTN 1-bp deletion mutation in exon 335, a critical alteration that leads to a frameshift mutation and results in truncated A-band titin, a hallmark of DCM. They ingeniously designed two specific AONs to selectively mask TTN exon 335 and unequivocally demonstrated that exon skipping was achieved without disrupting the essential TTN reading frame. Moreover, comprehensive evaluation of AON-treated cardiomyocytes revealed remarkable findings: these cells maintained critical functions, including store-operated calcium entry and fractional shortening, while also preserving sarcomeric formation when compared to control samples. These compelling results highlight the substantial potential of the treated cardiomyocytes to uphold vital cellular function and structural integrity, powerfully showcasing their capacity to compensate for the loss of exon 335.

Arrhythmogenic right ventricular cardiomyopathy (ARVC; MIM #602087) is a devastating heart disease marked by the replacement of the myocardium—predominantly in the right ventricle—with fatty and fibrous tissue. This transformation can trigger dangerous cardiac arrhythmias and, in the most tragic cases, lead to sudden cardiac death. Remarkably, approximately fifty percent of all ARVC cases are familial and inherited in an autosomal dominant pattern, though they exhibit reduced penetrance. Importantly, the TTN gene, located near one of the 12 identified ARVC loci, has been sequenced in 38 patients with familial ARVC (Taylor et al., 2011). This research uncovered eight unique nonsynonymous variants of TTN in seven patients, with two of these variants found in a single individual with compound heterozygosity. A particularly significant mutation, c.8687C>T (p.Thr2896Ile), has shown a clear and compelling relationship with the phenotype in a large family. This variant is predicted to hurt the highly conserved immunoglobulin-like domain 10 located in the I-band of TTN (Taylor et al., 2011). It is hypothesized that this mutation may compromise the structural integrity of the I-Ig10 domain, making it more vulnerable to proteolysis. This step is believed to be a critical precursor in the disease mechanism that ultimately leads to the emergence of the ARVC phenotype (Mallat et al., 1996).

There are four critical TTN mutations linked to familial hypertrophic cardiomyopathy (HCM; MIM #613765), a condition that stands as the leading cause of sudden cardiac death among young adults (Frey et al., 2012). HCM is defined by significant hypertrophy of cardiomyocytes, especially affecting the interventricular septum, alongside diastolic dysfunction of the cardiac ventricles, which is aggravated by fibrosis and myofibrillar disarray (Arimura et al., 2009). While the precise etiologies of HCM remain partially elusive, existing evidence indicates that only 50%–70% of familial HCM cases conform to an autosomal-dominant inheritance pattern. Over 20 different genes have been implicated in HCM, though they account for only a subset of affected individuals. Most of the mutations target proteins within the cardiac sarcomere, with the majority found in the MYH7 gene (encoding beta myosin heavy chain) and MYBPC3 gene (encoding MyBPC) (Frey et al., 2012). The four TTN mutations linked to HCM are particularly significant, as they are autosomal-dominant missense mutations that yield various gain-of-function effects. One notable Z-line mutation (c.2219G>T, p. Arg740Leu) markedly increases the binding affinity to α-actinin (Satoh et al., 1999). Another mutation in the cardiac-specific N2B exon 49 (c.12347C>A, p. Ser4116Tyr) enhances the interaction between TTN and DRAL/FHL2 (Matsumoto et al., 2005). Furthermore, two mutations in exons 103 and 104 of the N2A region (c.29231G>A, p. Arg9744, and c.29543G>A, p. Arg9848Gln) strengthen the critical interaction between TTN and T-CARP (Arimura et al., 2009).

Monogenic restrictive cardiomyopathy (RCM) is a rare but significant form of inherited cardiomyopathy, marked by a reduction in diastolic volume of one or both ventricles while maintaining normal or near-normal systolic function and wall thickness. This unique condition makes up the least common category of inherited heart disorders. To date, only a handful of mutations have been identified as causative for RCM (Peled et al., 2014). Recent research on a Sephardic family has unveiled a dominant missense mutation that impacts a conserved residue within the fibronectin-3 domain at the A/I junction, specifically identified as [c.50057A>G, p. Tyr16686Cys].

## 4 Conclusion

As the largest known protein in the human body, TTN has a crucial, yet not entirely understood, role in cardiac physiology and the pathogenesis of cardiovascular diseases. Ongoing research continues to reveal the essential roles of TTN in myofibril assembly, structural stabilization, and intracellular signal transduction. Despite this knowledge, crucial questions linger regarding the gene’s role in cardiomyopathies and the broader landscape of heart disease. Is it inevitable that all patients with TTN mutations will experience cardiac changes leading to heart failure, or is a second “hit” from environmental, genetic, or epigenetic factors always required for this progression? Could genetic alterations within TTN serve as a susceptibility factor for these conditions or even for heart failure at large? With the rapid advancement and increasing accessibility of large-scale quantitative data approaches, we stand on the brink of uncovering answers to these pressing questions. Such revelations promise to deepen our understanding of the pivotal role TTN plays in both health and disease. Understanding how disruptions in these essential processes can lead to cardiac dysfunction and serious human diseases is fundamental to advancing our knowledge and treatment of these conditions. Notably, alterations in TTN represent a valuable opportunity for developing targeted therapies for both genetic and acquired cardiomyopathies, offering hope for improved treatment options in these conditions.
